# Unusual shape-preserved pathway of a core-shell phase transition triggered by orientational disorder

**DOI:** 10.1107/S2052252522011034

**Published:** 2023-01-01

**Authors:** Mengya Li, Weiwei Tang, Junbo Gong

**Affiliations:** a School of Chemical Engineering and Technology, State Key Laboratory of Chemical Engineering, Tianjin University, Tianjin 300072, People’s Republic of China; b Collaborative Innovation Center of Chemistry Science and Engineering, Tianjin 300072, People’s Republic of China; c Key Laboratory Modern Drug Delivery and High Efficiency, Tianjin University, Tianjin 300072, People’s Republic of China; Sun Yat-Sen University, China

**Keywords:** crystal defects, core-shell structure, hydrates, uric acid

## Abstract

The ubiquitous presence of crystal defects provides great potential and opportunities to construct the desired structure (hence with the desired properties) and tailor the synthetic process of crystalline materials. However, little is known about their regulation role in phase transition and crystallization pathways. It was generally thought that a phase transition in solution proceeds predominantly via the solvent-mediated phase-transformation pathway due to energetically high-cost solid-state phase transitions (if any). Herein, we report an unprecedented finding that an orientational disorder defect present in the crystal structure triggers an unusual pathway of a core-shell phase transition with apparent shape-preserved evolution. In the pathway, the solid-state dehydration phase transition occurs inside the crystal prior to its competitive transformation approach mediated by solvent, forming an unconventional core-shell structure. Through a series of combined experimental and computational techniques, we revealed that the presence of crystal defects, introduced by urate tautomerism over the course of crystallization, elevates the metastability of uric acid dihydrate (UAD) crystals and triggers UAD dehydration to the uric acid anhydrate (UAA) phase in the crystal core which precedes with surface dissolution of the shell UAD crystal and recrystallization of the core phase. This unique phase transition could also be related to defect density, which appears to be influenced by the thickness of UAD crystals and crystallization driving force. The discovery of an unusual pathway of the core-shell phase transition suggests that the solid-state phase transition is not necessarily slower than the solvent-mediated phase transformation in solution and provides an alternative approach to constructing the core-shell structure. Moreover, the fundamental role of orientational disorder defects on the phase transition identified in this study demonstrates the feasibility to tailor phase transition and crystallization pathways by strategically importing crystal defects, which has broad applications in crystal engineering.

## Abstract 2

Orientational disorder defects present in the crystal structure of uric acid dihydrate trigger a solid-state dehydration phase transition inside the crystal prior to the competitive transformation mediated by solvent, forming an unconventional core-shell structure.

## 1. Introduction

Multicomponent assembly and crystallization play important roles in synthetic materials (Jiang *et al.*, 2020 ▸), biology (Gal *et al.*, 2016 ▸), geology (Zidarov *et al.*, 2001 ▸) and extraterrestrial systems (Greenwood *et al.*, 2008 ▸); for example, the formation of hierarchically organized structures and the morphology of polydisperse gold thiol­ate nanoplatelets (Jiang *et al.*, 2020 ▸), sea urchins (Towe, 2006 ▸) and coccoliths (Gal *et al.*, 2016 ▸) often involve multiple complex pathways (Korevaar *et al.*, 2012 ▸; Boekhoven *et al.*, 2015 ▸). In the system, multiple kinetic and thermodynamic events are often competitive with each other, which require external or internal ‘triggers’ to direct the system towards the selection of a certain pathway (Pappas *et al.*, 2015 ▸; van Rossum *et al.*, 2017 ▸; Van Driessche *et al.*, 2021 ▸). These triggers can be external physical or chemical factors such as the wide use of solvents (Yuan *et al.*, 2008 ▸; Abbas *et al.*, 2007 ▸), modifiers (Zhang *et al.*, 1996 ▸; Chen & Rosi, 2010 ▸), and/or templates (Archibald & Mann, 1993 ▸) that direct polymorphic crystallization (Sommerdijk & Sleutel, 2018 ▸) or amorphous-to-crystalline phase-transition pathways (Zhou *et al.*, 2021 ▸). A recent study identified an internal trigger, *i.e.* lattice strain (Huang *et al.*, 2021 ▸), which is accumulated over the growth course of a layered crystal, for a ‘spontaneous’ crystal transition from a layered crystal to a pillared-layer crystal in the crystallization from a multicomponent environment. Although crystal defects are considered to be universally present in crystalline materials, their application in the regulation of phase-transition pathways is rare, in particular for organic crystals.

Here we report that the stacking defect produced during crystallization serves as an ‘internal trigger’ for pathway selection of the phase transition in a multicomponent crystallization system. While optimizing the conditions for the crystal synthesis of uric acid dihydrate (UAD) from a mixture of uric acid, hydro­chloric acid, water and NaCl at pH 5–6, we noticed the selective crystallization of a rectangular plate UAD crystal. After a while, a core phase identified as uric acid anhydrate (UAA) spontaneously formed inside the mother UAD crystal, leading to an unconventional core-shell structure. The core UAA crystal then broke through the shell undergoing surface local dissolution and recrystallized to a uniform crystal phase. Note that a series of events involved in this phase transition via the core-shell structure occurred spontaneously in the absence of an external trigger. Uric acid (UA) is the biological product of purine metabolism in mammals, but excessive UA concentration in acidic urine leads to the pathological formation of kidney stones, of which UAA and UAD are common organic components (Ringertz, 1966 ▸; Parkin & Hope, 1998 ▸). The metastable phase UAD can spontaneously transform to UAA through either solid-state dehydration in air or solvent-mediated phase transformation in aqueous solution (Zellelow *et al.*, 2010*a* ▸; Presores & Swift, 2014 ▸). A recent study by Swift and coworkers suggested that the defects induced by trace amounts of 2,4-di­amino­pyrimidine may impact the kinetics of the solid-state dehydration transition from UAD to UAA (Thornton *et al.*, 2020 ▸). However, the unique core-shell phase transition reported in this work was not observed in any of these investigations.

The phase transition between crystalline phases could have different pathways such as a solid-state phase transition (Yang *et al.*, 2019 ▸; Maher *et al.*, 2012 ▸), a solvent-mediated phase transformation (O’Mahony *et al.*, 2012 ▸; Tang *et al.*, 2020 ▸, 2021 ▸) and a vapor-mediated phase transition (Smets *et al.*, 2018 ▸); the first two are the most encountered in a solution crystallization system. Solid-state phase transition refers to the transition between solids: a spontaneous transition from a metastable phase and a stable phase in the absence of solvent due to the thermodynamic stability of two distinct structures (Yang *et al.*, 2019 ▸), involving a metastable crystal structure rearranging into a more stable phase (Maher *et al.*, 2012 ▸). This can be achieved through a cooperative single-crystal-to-single-crystal phase-transition pathway via collective and rapid propagation of molecules when polymorphs respond to external light or thermal stimulus (Sahoo *et al.*, 2013 ▸; Karothu *et al.*, 2016 ▸; Liu *et al.*, 2017 ▸) or more commonly a nucleation and growth mechanism (Shi *et al.*, 2021 ▸). Previously, we have demonstrated that both pathways can be influenced by the presence of microscopic crystal defects (Shi *et al.*, 2021 ▸). Crystal defects have been widely observed in a variety of crystalline materials from inorganic to organic compounds such as photoelectric materials (Demortière *et al.*, 2018 ▸), perovskites (Li *et al.*, 2021 ▸), pharmaceuticals (Yu *et al.*, 2020 ▸), metals (He *et al.*, 2017 ▸) and ceramics (Wang *et al.*, 2017 ▸). These defects display various types including (sub-)grain boundaries (Demortière *et al.*, 2018 ▸; Gong & Kelley, 2015 ▸; Bak *et al.*, 2020 ▸), dislocations (He *et al.*, 2017 ▸; Frolov *et al.*, 2013 ▸; Šmilauerová *et al.*, 2021 ▸), vacancies (Li *et al.*, 2021 ▸), impurity doping (Wang *et al.*, 2017 ▸) *etc.* which may lead to the appearance of lattice distortion and high distortion energy (Beyerlein *et al.*, 2015 ▸). Although the influence of microscopic crystal defects presented in inorganic or metal–organic materials has been extensively examined (Li *et al.*, 2021 ▸; Šmilauerová *et al.*, 2021 ▸), their effect on phase transition of organic crystals remains to be understood. It was suggested that the large fluctuation of energy, structure and composition at (or near) crystal defects may promote the nucleation of a new phase (Mnyukh, 2010 ▸). More recently, the variations of molecular diffusivity and stress relaxation near defect microdomains were also proposed to impact solid-state phase-transition kinetics (Smets *et al.*, 2020 ▸; Naumov *et al.*, 2015 ▸). But these studies do not reveal the impact of stacking defects on phase-transition pathways.

Solvent-mediated phase transformation is the mostly encountered phase-transition pathway in solution wherein the metastable phase dissolves accompanied by nucleation and growth of a more stable crystal (O’Mahony *et al.*, 2012 ▸; Tang *et al.*, 2020 ▸, 2021 ▸). It is generally thought that the solvent-mediated phase-transition pathway predominates in solution and forms prior to its ‘competitor’ solid-state phase transition (if any) (Li *et al.*, 2020 ▸; O’Mahony *et al.*, 2013 ▸; Budhysutanto *et al.*, 2010 ▸). Nevertheless, in this study we found that orientational disorder defects functioning as internal triggers can lead to the occurrence of a solid-state dehydration phase transition prior to the solvent-mediated phase-transformation pathway. The crystallization and phase-transition pathways were monitored *in situ* using an inverted microscope, and structure evolutions were examined by powder X-ray diffraction (PXRD), fourier transform infrared (FTIR) spectroscopy, transmission electron microscopy (TEM) and thermogravimetric analysis (TGA). The unique formation of the core-shell structure was confirmed by scanning electron microscopy (SEM) and micro-Raman spectroscopy. Crystal defects (*i.e.* orientational disorder) were unveiled by combined experimental and computational techniques including atomic force microscopy (AFM), single-crystal X-ray diffraction (SCXRD) and lattice energy calculations.

## 2. Results and discussion

### 2.1. Unusual core-shell structure and shape-preserved evolution

**Figure 1:**
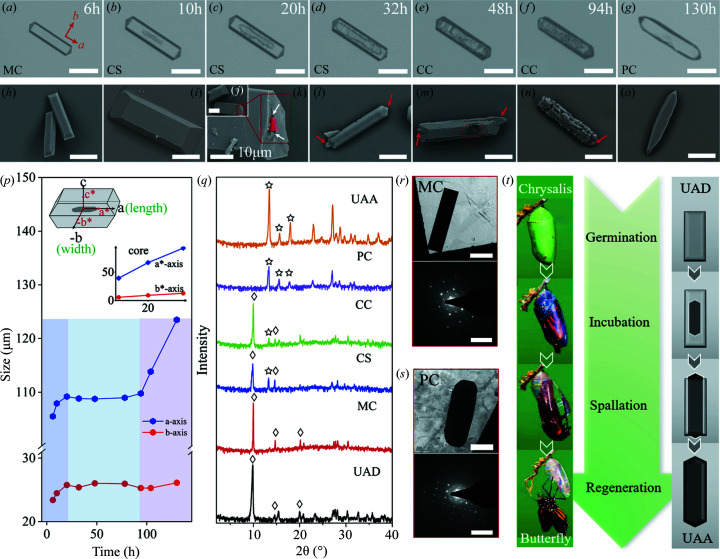
Morphology and structural evolutionary processes of the core-shell phase transition. (*a*)–(*g*) Time-resolved micrographs of a crystal crystallized from a solution containing 6.5 m*M* UA, hydro­chloric acid, water and 0.14 *M* NaCl at pH 5.5. The scale bar is 50 µm. MC – mother crystal, CS – core-shell crystal, CC –complex crystal, PC – pure crystal. (*h*)–(*o*) SEM images of the crystal isolated at different stages. The scale bar is 25 µm. (*p*) Size evolution of the length and width of the shell phase (inset, upper left). Schematic representation of a core-shell crystal (inset, upper right). The size evolution of the length and width of the core phase. (*q*) PXRD patterns of the crystal obtained at different stages. (*r*) and (*s*) TEM images and SAED patterns of MC initially formed and the resultant PC. The scale bar is 400 nm in the TEM images and 5 nm^−1^ in the SAED patterns. (*t*) Schematic illustration of the morphology evolution of the crystal at different stages.

The spontaneous crystallization of UA was monitored under an inverted-optical microscope from a multicomponent aqueous solution containing excess UA, hydro­chloric acid and 0.14 *M* NaCl at pH 5.5 at 37°C. Surprisingly, we discovered unusual evolutions of structure and morphology. A rectangular-shaped crystal [Figs. 1 ▸(*a*) and 1(*h*)] was firstly crystallized and observed at ∼6 h, and the crystal continuously grew with the depletion of UA in solution [Figs. 1 ▸(*b*), 1(*c*) and 1(*p*)], named the mother crystal (MC). Unexpectedly, a new phase apparently presenting as a ‘macro defect’ appeared inside the crystal at 10 h [Fig. 1 ▸(*b*)]. SEM images of the crystal [Figs. 1 ▸(*i*) and 1(*j*)] further confirm the new phase located at the core of the rectangular-shaped crystal [highlighted in red in Fig. 1 ▸(*k*)], not on the surface [smooth surface in Figs. 1 ▸(*h*) and 1(*i*)], behaves like a core-shell crystal (CS). Time-elapsed micrographs allowed us to track the evolution of the core phase. We observed gradual spreading of the core phase in crystal from the center to tips along the a* axis [Figs. 1 ▸(*c*) and 1(*d*) and 1 ▸(*p*), inset], finally growing out of the shell crystal [red arrows in Fig. 1 ▸(*l*)] with a slower spreading rate along the b* axis [Fig. 1 ▸(*p*), inset]. Although the shell crystal displays fast growth at the beginning (*i.e.* 6–20 h), it stopped growing in the time period between 20 and 94 h. Note that the new core phase appears within the growth period of the shell crystal, suggesting that the growth solution remains supersaturated with both core and shell phases. The surface of shell crystal then became darker [Figs. 1 ▸(*e*) and 1(*f*)] and macroscopically rough [Figs. 1 ▸(*m*) and 1(*n*)] at 48 h, but the entire crystal still maintained the rectangular shape with identical length and width, indicating only local dissolution and re-crystallization, named complex crystals (CC). The boundary between core and shell phases is indistinguishable at 48–94 h [Fig. 1 ▸(*f*)]. Finally, crystal facets evolved to be smooth again [Figs. 1 ▸(*g*) and 1(*o*)], and this new crystal grew into a rectangular shape similar to the initial crystal habit formed though with a higher anisotropic growth rate [Fig. 1 ▸(*p*)]. The full time-elapsed evolution of crystal morphology was recorded, see Movie S3 of the supporting information. Although we did not clearly observe the transportation pathway of internal water molecules released during dehydration, this may be due to the resolution limitation of optical microscopes, it is possible that the internal water molecules were evacuated and stored in the narrow gaps [indicated by white arrows in Fig. 1 ▸(*k*)] between the core and the shell phases.

The crystals obtained at different growth stages were extracted and analyzed by PXRD, as shown in Fig. 1 ▸(*q*). We found that the initial MCs formed are UAD whereas the characteristic peaks of UAA at 13.49, 15.70 and 18.04° gradually intensify over time, corresponding to the growth of core phase in the MC [Figs. 1 ▸(*b*)–1(*d*)] and the dissolution of the shell crystal [Figs. 1 ▸(*e*) and 1(*f*)]. Finally, the irregular crystal with a smooth surface was found to be the pure UAA phase, named the pure crystal (PC). The results thus demonstrated a phase transition from UAD to UAA with an unusual shape-preserved pathway. The initially MC (shell phase) and the resultant PC (core phase) after the phase transition were characterized by TEM [Figs. 1 ▸(*r*) and 1(*s*)]. The clear, well separated diffraction spots in the selected area electron diffraction (SAED) analysis confirm the single crystallinity of the UAD and UAA phases for the shell and core crystals, respectively.

Our observed, unusual core-shell evolution of single crystals depicts a phase transition from the UAD to the UAA form. In contrast to the most commonly observed solvent-mediated phase-transformation pathway through the first dissolution of a metastable form and recrystallization of the more stable form (Presores & Swift, 2014 ▸), the core-shell phase-transition pathway starts from the interior of the UAD MC and occurs in a supersaturated solution relative to the metastable UAD form, behaving like the metamorphosis of a butterfly, schematically illustrated in Fig. 1 ▸(*t*). Similarly, the core-shell phase-transition process may be described by four stages as follows: (1) the onset formation of a metastable, rectangular UAD crystal behaving as the MC at the germination stage; (2) the core UAA phase forms in the interior of the MC and gradually spreads throughout the MC, presenting as a CS at the incubation stage; (3) the core UAA phase grows out from tips of the MC and grows continuously by local surface dissolution of the shell UAD crystal, just like CC, at the spallation stage; (4) growth of the core crystal of the pure UAA phase (PC) with smooth facets at the regeneration stages.

**Figure 2:**
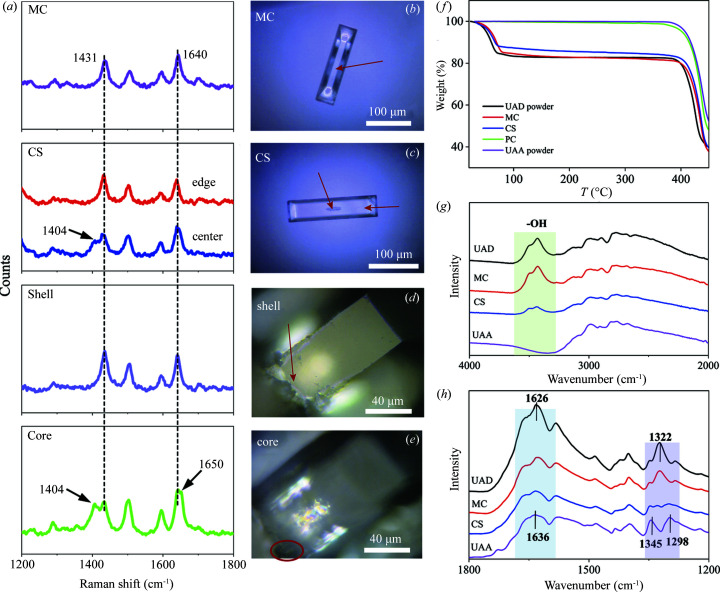
Structure and interactions of the core and shell phases. (*a*) Spatial-resolved micro-Raman spectra of the crystals obtained at different stages of the morphology evolution. Micrographs of the (*b*) MC and (*c*) CS. (*d*) and (*e*) Cross-section of CS showing the (*d*) shell phase and the (*e*) core phase. (*f*) TGA plots of crystals obtained at different evolution stages. (*g*) and (*h*) FTIR spectra of crystals obtained at different stages. The green region in (*g*) highlighted the —OH stretching of water molecules, and the blue and purple regions in (*h*) highlighted the H—O—H symmetric bending vibrations and C—N stretching, respectively.

We further employed spatial-resolved micro-Raman spectroscopy to characterize the detailed structure of the crystal at different locations in different stages of evolution, and the results are illustrated in Fig. 2 ▸. The Raman spectrum of UAA crystals is similar to that of UAD (Fig. S1 of the supporting information), but two characteristic peaks can be identified in the range 1200–1800 cm^−1^ where UAD displays characteristic peaks at 1431 and 1640 cm^−1^, but these two bands shift to 1404 and 1650 cm^−1^, respectively, in UAA (Thornton *et al.*, 2020 ▸). We found the MC initially formed [Fig. 2 ▸(*b*)] at the germination stage appears homogeneous, and the representative spectrum was recorded at the site located in the center of the MC [red arrow in Fig. 2 ▸(*b*)], showing two characteristic peaks at 1431 and 1640 cm^−1^ which correspond to the UAD form [Fig. 2 ▸(*a*)]. However, the CS [Fig. 2 ▸(*c*)] displays the inhomogeneous feature at the center (core phase) of the crystal compared with the edge phase [red arrows in Fig. 2 ▸(*c*)]. The Raman spectra reveal the presence of the UAA phase characterized by the appearance of an additional shoulder peak at 1404 cm^−1^ compared with the UAD phase (the presence of 1431 and 1640 cm^−1^ peaks) at the edge. Note that the UAA phase originated from the core of the UAD crystal rather than the surface. To further demonstrate this point, we cut CS near the center region and measured directly both the shell and the core phases [Figs. 2 ▸(*d*), and 2(*e*)]. As expected, the shell phase displays identical peaks to those of UAD, whereas the core phase presents two strong peaks at 1404 and 1650 cm^−1^ corresponding to the UAA phase. In addition, the Raman spectra of CC at the spallation stage and PC at the regeneration stage were also collected (Fig. S2) and show the characteristic peaks of UAD (1431 and 1640 cm^−1^) and UAA (1404 and 1650 cm^−1^), respectively, which agree well with the aforementioned structure evolution [Fig. 1 ▸(*q*)].

TGA was also performed to confirm the phase transition of UAD to UAA, as illustrated in Fig. 2 ▸(*f*). The water molecules occupy 17.6% mass fraction in the UAD crystal. The TGA curve of MC displays the loss of 17.2% (mass fraction) water in the range 30–200°C, which is close to that of the UAD powder (17.3%). Both values are close to the theoretical value of 17.6% water loss in the UAD crystal, confirming MC in the UAD form. In contrast, the TGA curve of CS at the incubation stage presents 14.4% loss of water, which is equal to the water loss of UAD crystals containing 21% (mole fraction) UAA phase in the crystal core. The PC obtained at the regeneration stage shows no water loss in the same temperature range with chemical degradation presenting at above 350°C, demonstrating pure UAA phase. Moreover, FTIR spectroscopy was employed to characterize the structure of UA crystals at different stages. As seen in Fig. 2 ▸(*g*), the FTIR spectrum of MC exhibits OH peaks of H_2_O at 3300–3600 cm^−1^, identical in both intensity and position to that of UAD. When the UAA phase formed in the core of the UAD crystal (CS), the peak intensity decreased due to the reduced number of water molecules in the crystal. The characteristic peaks highlighted at 1200–1800 cm^−1^ also confirm the phase transition with the presence of the core UAA phase in Fig. 2 ▸(*h*).

### 2.2. Crystallization kinetics of core-shell crystals

**Figure 3:**
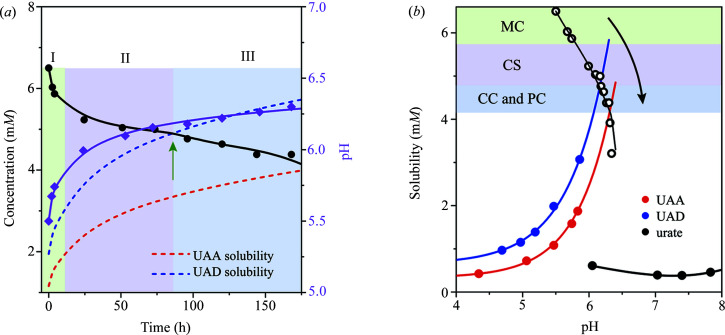
Crystallization kinetic profiles and phase diagram. (*a*) Time-resolved depletion profile of the urate concentration (black circles) and the corresponding pH (pink circles) evolution profile in solution crystallized from a 6.5 m*M* urate concentration at an initial pH of 5.5. The lines are interpolated to guide the eye. The blue-dashed line represents the solubility of UAD measured and interpolated from experimental data (Fig. S4). The red-dashed line represents the solubility of UAA measured and interpolated from experimental data (Fig. S4). (*b*) Phase diagram of the urate crystallization with respect to solution pH. The black, red and blue points are the measured solubilities of urate sodium monohydrate, UAA and UAD, respectively, and the corresponding lines are interpolated to guide the eye. The black empty circles represent the time-resolved depletion profile of the urate concentration, and the corresponding line is interpolated to guide the eye, representing the dynamic operating line over time.

UA is a polyprotic acid with two dissociation constants [p*K*_a_ = 5.4 and 10.3 (Kahn *et al.*, 1997 ▸; Smith *et al.*, 1988 ▸)] and presents predominantly from the neutral to monovalent species with pH increasing in the range 5–7 (Fig. S3). Under these pH conditions, the crystallization of UA solutes reduces the acidic proton in solution, leading to the concomitant increase of pH and speciation population toward to the monovalent form. To account for this influence, we thus monitored the changes in both pH and concentration of UA in solution over time. The time-resolved concentration depletion profile of urate in solution with the corresponding evolution of pH was shown in Fig. 3 ▸(*a*), and the kinetic profiles of the crystallization process are described in three stages (I–III).

In stage I, the nucleation and growth of MC (in UAD form) lead to the rapid depletion of the concentration of UA in solution and the concomitant increase of pH within 6 h. The MC then continuously grew to a large size and formed the core phase (CS) in the MC but with slower kinetics of solute depletion in stage II, due to the approach to the solubility limit [blue-dashed line in Fig. 3 ▸(*a*) and blue line in Fig. 3 ▸(*b*)]. This was also reflected in solution pH evolution to level-off. Note that the concentration of UA at 85 h corresponds to the equilibrium solubility of the UAD form. In stage III, UA solutes are under-saturated relative to metastable UAD crystals but still supersaturated with the stable UAA phase [red-dashed line in Fig. 3 ▸(*a*) and red line in Fig. 3 ▸(*b*)], resulting in the dissolution of the MC shell, appearing in the form of CC [Figs. 1 ▸(*e*), 1(*f*), 1(*m*) and (*n*)]. When the UAA phase grew out of UAD crystal shell, the surface growth of the UAA crystal takes the predominant role of forming PC with smooth facets in a ‘self-healing’ manner. The growth rate of the UAA core crystal is probably greater than the dissolution rate of the UAD shell crystal, as revealed by continuously decreasing of urate concentration over time. This is also consistent with observations of local dissolution and rough features in Fig. 1 ▸. Growth regimes of UA crystals at different stages, presented as the form of MC, CS, CC or PC, can be clearly described in the phase diagram presented in Fig. 3 ▸(*b*). Solubility data of UAD and UAA were determined and are given in Fig. S4. It is evident that the dynamic operating line [black arrow in Fig. 3 ▸(*b*)] starts from an initial concentration point far from equilibrium where the spontaneous crystallization occurred and MC is formed. Then, growth of MC accompanies the formation of a new UAA phase in the core of the crystal, presented as CS, and solute concentration approaches the equilibrium solubility boundary of UAD. Finally, the solute concentration decreases across the solubility of UAD and continuously approaches the solubility boundary of the UAA phase, leading to the dissolution of UAD shell crystals presented as CC and the resultant form of PC (*i.e.* pure stable UAA form). Note that the crystallization of metastable UAD crystals from our growth condition (6.5 m*M* UA) is consistent with previous reports in the literature (Presores & Swift, 2014 ▸; Zellelow *et al.*, 2010*a* ▸,*b* ▸).

### 2.3. Core-shell phase transition in air

Our observations suggest that the new stable phase formed in the core of the crystal and then spread across the interior of the crystal, likely by a solid-state phase transition. The solvent-mediated phase transformation has been commonly observed in solution crystallization, but this must be not the case here for two reasons. Firstly, we observed that the phase transition occurs in the core or interior of the crystal, not on the surface. More evidently, the onset transition of the core phase is accompanied by the growth of UAD crystals, suggesting the solution is still supersaturated relative to both the UAD and the UAA phases.

**Figure 4:**
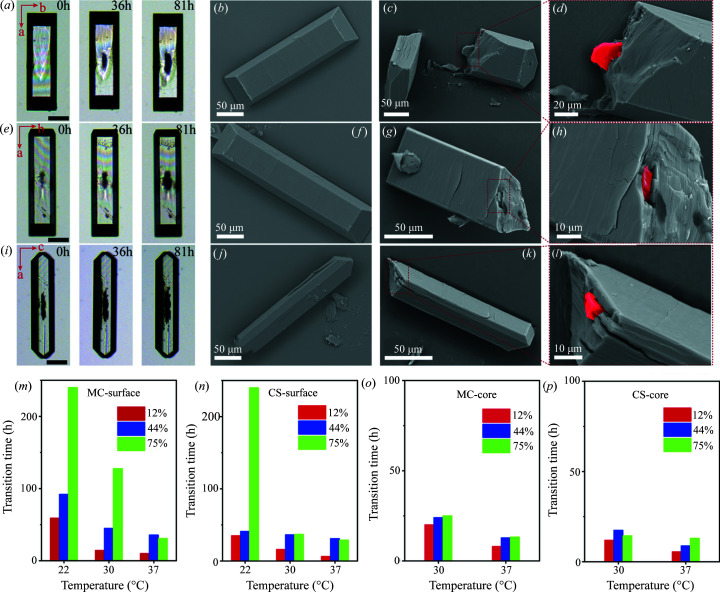
Core-shell phase transition in air. Morphology evolutions of (*a*) MC and [(*e*), top view; (*i*), side view] CS in air at 37°C and RH 75%. The scale bar is 50 µm. SEM images of surface and cross-section of (*b*)–(*d*) MC, (*f*)–(*h*) and (*j*)–(*l*) CS selected after 36 h. The onset transition time of (*m*), (*o*) MC and (*n*), (*p*) CS in air at temperatures from 22 to 37°C and RHs ranging from 12 to 75%.

In order to test our solid-state phase-transition hypothesis, we investigated the phase-transition behavior of crystals (*i.e.* MC and CS) in air at temperatures ranging from 22 to 37°C and relative humidity (RH) ranging from 12 to 75%. The morphological evolution of MC and CS in air at 37°C and RH 75% were recorded, and the results are shown in Fig. 4 ▸. We observed similar results that the core of the MC firstly became darker at about 36 h and continuously grew from the center to the tip of the crystal predominantly along the *a* axis [Fig. 4 ▸(*a*)], albeit with even slower kinetics. SEM images clearly demonstrated the smooth feature of the MC surface [Fig. 4 ▸(*b*)] after the appearance of a new phase in the core, as revealed in the cross-section of the crystal where a new crystal protrusion formed [Figs. 4 ▸(*c*) and 4(*d*), the new phase is highlighted in red]. Furthermore, we also observed the growth of CS (already presented with the new UAA phase in the core) over time in air [Fig. 4 ▸(*e*)], similar to that in solution. When the same CS was observed [shown in Fig. 4 ▸(*f*)] from the side direction [perpendicular view to that in Fig. 4 ▸(*e*)], we still observed the core phase located near the center of the crystal. SEM images again show the feature of a smooth surface viewed from two perpendicular directions [Figs. 4 ▸(*f*) and 4(*j*)]. The cross-section of these crystals clearly shows the presence of new crystal phase formed in the interior of the CS highlighted in red [Figs. 4 ▸(*g*), 4(*h*), 4(*k*) and 4(*l*)]. Collectively, these support the core-shell structure of CS with the formation and growth of the UAA phase in the core under air. Similar results were also observed under two other RH conditions, 12 and 44%, while the time for core-shell phase transition varies (Fig. S5).

We examined the influences of temperature and RH on the rate of phase transition of MC and CS both on the surface and in the core by measuring and defining the time of onset phase transition. The measurement of onset transition time from the core-shell structure of CS samples is difficult and hard to distinguish the new born core phase from the one presented, but we found that the dimension of the CS core phase increases rapidly when the phase transition is triggered (Fig. S6). We thus defined the turning point as the time of onset phase transition of CS. The results are illustrated in Figs. 4 ▸(*m*)–4(*p*). Interestingly, it was found that the time for onset transition of the core was predominantly controlled by temperature, and the effect of humidity was nearly negligible [Figs. 4 ▸(*o*) and 4(*p*)]. But it is evident that the time for the phase transition occurring in the core is less than that on the surface at 30 and 37°C, which mimics the first appearance of the new phase in the core of the crystal in solution crystallization. When the phase transition occurred on the crystal surface, we found the transition time of either MC or CS is significantly reduced by increasing temperature and reducing RH [Figs. 4 ▸(*m*) and 4(*n*)]. The accelerating phase transition at higher temperature is expected due to the reduced energy barrier for dehydration of water, regardless of the surface or the core. On the contrary, moisture in the environment may significantly affect the dehydration of UAD to UAA in the surface because the absorption of water molecules is expected on the crystal surface which hinders the process of dehydration. Therefore, both temperature and humidity determine the procedure of phase transition on the crystal surface (Zellelow *et al.*, 2010*c* ▸). However, only temperature will affect the speed of phase transition in the core.

### 2.4. Crystallization regime of crystals with defects via core-shell phase transition

**Figure 5:**
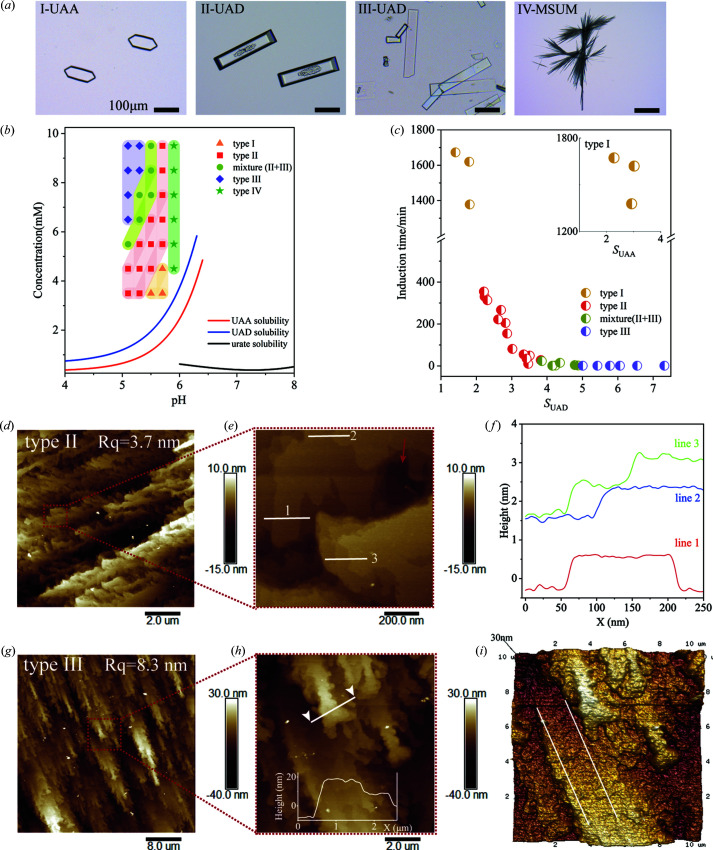
Crystallization regime of core-shell crystals. (*a*) Four types of urate crystals formed under various crystallization conditions. (*b*) Crystallization map of four types of urate crystals. The spots of different colors represent different crystal types, the shadows with corresponding colors are interpolated to guide the eye. (*c*) Induction time distribution for crystallization of different types of crystals as a function of solution super-saturation (relative to the UAD phase). Inset represents the induction time distribution for a type-I crystal plotted as super-saturation relative to the UAA phase. (*d*) and (*e*) Topology of the basal (001) surface of the type-II crystal and (*f*) step height profiles marked by white lines in (*e*). (*g*) and (*h*) Topology of the basal (001) surface of the type-III crystal and (*i*) 3D view of the (001) surface.

We expanded crystallization conditions of UA to identify the formation conditions of crystals with defects displaying the core-shell phase-transition pathway. More than 30 crystallization experiments were performed using a series of urate concentrations and solution pHs, corresponding to different supersaturations (Table S2). Four types of crystals were obtained, marked I–IV in Fig. 5 ▸(*a*), and summarized in Table S1 of the supporting information. The type-I crystal displays a hexagonal shape crystallized from a lower supersaturated solution and the UAA phase was confirmed by micro-Raman spectroscopy (Fig. S7). Type II is a representative of CS (mixture of UAD and UAA) and crystallized in a medium level of supersaturation. Type III is also UAD (Fig. S7) but exhibits a thin plate from a high supersaturated solution. Note that no UAA phase was detected in type-III crystals. Type IV is sodium urate monohydrate, where urate crystallizes as a monovalent species (Fig. S3).

We found that three types of UA crystals are closely related to their initial crystallization supersaturations [Fig. 5 ▸(*b*)], displaying a distinct induction time [Fig. 5 ▸(*c*)]. Crystallization of UA molecules from a low supersaturation in solution leads to the formation of the stable UAA phase with an extremely long induction time consistent with previous reports in the literature (Zellelow *et al.*, 2010*a* ▸,*b* ▸). As the supersaturation increased up to 2.0, we found that UA crystallized as the UAD phase exhibiting two types, a core-shell ‘defected’ crystal (type II) and a thin plate (type III). They apparently differ in crystal morphology, especially in the thickness of crystal, due to kinetic differences in nucleation and crystal growth rates. A higher level of solution supersaturation would induce a number of UAD nuclei that form in minutes [Fig. 5 ▸(*c*)], leading to fast depletion of solutes in solution and growth of thin plate crystals (type III). Yet UA nucleated more slowly at a moderate level of supersaturation on the order of hours [Fig. 5 ▸(*c*)], and a small number of crystals formed and grew into thick-plate UAD crystals with the core phase inside (type II).

Furthermore, we examined the surface feature of type-II and type-III crystals by AFM to identify their structure and morphology difference [Figs. 5 ▸(*d*)–5(*i*)]. The feature of step bunching is presented on the basal (001) surface of both type-II and type-III crystals [Fig. 5 ▸(*d*) and 5(*g*)], but the roughness, *R*_q_, of the type-III crystal (*R*_q_ = 8.3 nm) on a 10 × 10 µm scale is remarkably greater than that of type II (*R*_q_ = 3.7 nm). From Figs. 5 ▸(*d*) and 5(*e*), type II displays no continuous terraces with locally smooth surfaces, and the step height [marked by white lines in Fig. 5 ▸(*e*)] is about 0.63–0.98 nm [Fig. 5 ▸(*f*)], closed to half the *c* axis (17.45 Å) of the unit cell (Parkin & Hope, 1998 ▸). This suggests the growth mode of type II by island generation and layer spreading. In addition, deep-pit defects were also observed [indicated by the red arrow in Fig. 5 ▸(*e*); the depth of the pit was 8.23 nm, nearly 8 layers]. However, the rougher type-III crystal surface demonstrated high density of step bunches [Fig. 5 ▸(*g*)] with narrow terraces [between the white lines in Fig. 5 ▸(*i*)]. The step height [marked in Fig. 5 ▸(*h*)] was about 26.67 nm, almost equal to 16 growth layers. The results indicate a transition of growth mechanism to step bunching growth (Enslin *et al.*, 2019 ▸; Olafson *et al.*, 2017 ▸; Durbin *et al.*, 1993 ▸; Gratz *et al.*, 1991 ▸; Jiang *et al.*, 2001 ▸).

**Figure 6:**
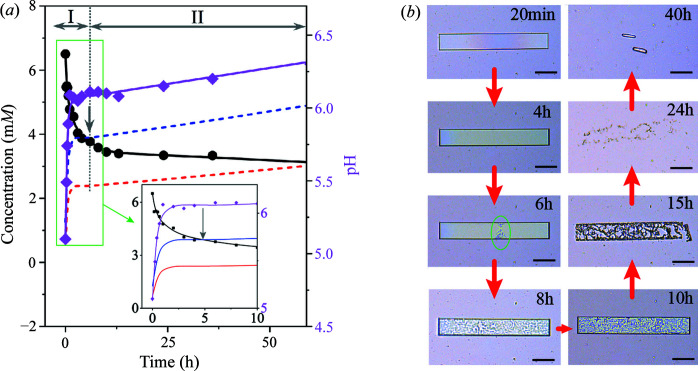
Crystallization kinetic profile of type-III crystals accompanied by a solvent-mediated phase transformation. (*a*) Time-resolved depletion profile of the urate concentration (black circles) and the corresponding pH (pink diamond) evolution profile in solution crystallized from a 6.5 m*M* urate concentration at an initial pH of 5.1. The lines are interpolated to guide the eye. I – crystallization of the UAD phase, II – solvent-mediated phase transformation from UAD to UAA. The blue- and red-dashed lines represent the solubility of UAD and UAA, respectively, measured and interpolated from experimental data (Fig. S4). The inset highlights the crystallization kinetic profile before 10 h. (*b*) Crystal morphology evolution of type-III crystals over time observed under an optical microscope. The scale bar is 50 µm.

Both type-II and type-III crystals are UAD phase, however, their pathways of phase transition from UAD to UAA are distinct in solution. We investigated the crystallization kinetics of type-III crystals (Fig. 6 ▸). A number of type-III crystal nuclei were formed in a few minutes in a high supersaturated solution (6.5 m*M* UA, initial pH 5.10) and then grown into thin plates [before 20 min, in Fig. 6 ▸(*b*)] corresponding to the sudden drop of urate concentration and rapid increase of solution pH over the course of crystallization [Fig. 6 ▸(*a*)]. Type-III thin crystals were then gradually dissolved from their surface [marked by the green circle at 6 h in Fig. 6 ▸(*b*)] due to urate concentration dropping below the solubility of UAD [Fig. 6 ▸(*a*)]. While type-III crystals dissolved [at 6–24 h in Fig. 6 ▸(*b*)], the new UAA phase was formed and grown from solution [at 40 h in Fig. 6 ▸(*b*)] through a typical pathway of solvent-mediated phase transition (Movie S4), which agrees well with the literature (Presores & Swift, 2014 ▸). The time taken for the solvent-mediated phase-transformation pathway from UAD to UAA (∼6 h) was found to be much shorter than that of the type-II core-shell phase transition (∼80 h).

The phase-transition behavior of type-III crystals in air is remarkably different from that of type II. The surface of type-III crystals first show a number of cracks and spots at 37°C and RH 12%, and then further cracks covered the surface owing to dehydration (Fig. S8). The onset transition time of type-III crystals in air is significantly reduced with increased temperature and decreased humidity (Fig. S9), suggesting both temperature and humidity in the environment controlled the course of the phase transition. This is similar to the observations during the phase transition on the surface of the type-II crystals but different from the transition that occured in the core. The time for the onset phase transition of type-III crystals is faster in general than that of type II (Table 1 ▸), but no core phase was detected throughout the phase-transition experiments. We estimated the activation energies (*E*_a_) of the solid-to-solid phase transitions for both type-II and type-III crystals using the non-isothermal method (see the supporting information for details, Figs. S10 and S11) (Zellelow *et al.*, 2010*c* ▸; Starink, 2003 ▸; Ozawa, 1992 ▸). The apparent activation energy of dehydration is estimated by the Kissinger–Akahira–Sunrose model to be 59.8 kJ mol^−1^ for type II and 55.3 kJ mol^−1^ for type III. They are 62.3 and 57.9 kJ mol^−1^ for type II and type III, respectively, when the Flynn–Wall–Ozawa model was employed. The result suggests type-II crystals are more difficult to dehydrate via the solid-state phase-transition pathway.

### 2.5. Mechanism of the core-shell phase transition

Why does the system select a more complex crystallization pathway through a core-shell phase transition and not the commonly observed solvent-mediated phase transformation? Both type-II and type-III crystals are the UAD phase but why do they favour different phase-transition pathways. What is the internal trigger for such a pathway selection? The precondition for the pathway of a core-shell phase transition is the initial formation of the core UAA phase via solid-state dehydration of UAD that occurs inside the shell crystal prior to solvent-mediated phase transformation in solution. The dehydration in the crystal interior is often considered to be more difficult than that on the surface because of the relatively large molecular mobility on the surface. Nonetheless, such common sense appears not applicable here. We found that the apparent activation energy of solid-state dehydration (∼60 kJ mol^−1^) of the UAD phase is significantly lower than the values reported (∼70–170 kJ mol^−1^) in the literature (Watts *et al.*, 2021 ▸; Takahashi & Uekusa, 2022 ▸), which indicates crystal defects may play an important role in the UAD-to-UAA solid-state phase transition. Such defect may be introduced in the growth of UAD crystals at nearly neutral pH (5–6), wherein monovalent tautomer species were suggested to be significant (Fig. S3) (Zellelow *et al.*, 2010*a* ▸; Kahn *et al.*, 1997 ▸; Jiménez & Alderete, 2005 ▸; Wang *et al.*, 2018 ▸). This structurally similar tautomer may serve as an ‘impurity’ to disrupt nucleation and growth kinetics, leading to the formation of type-II crystals with defects.

**Figure 7:**
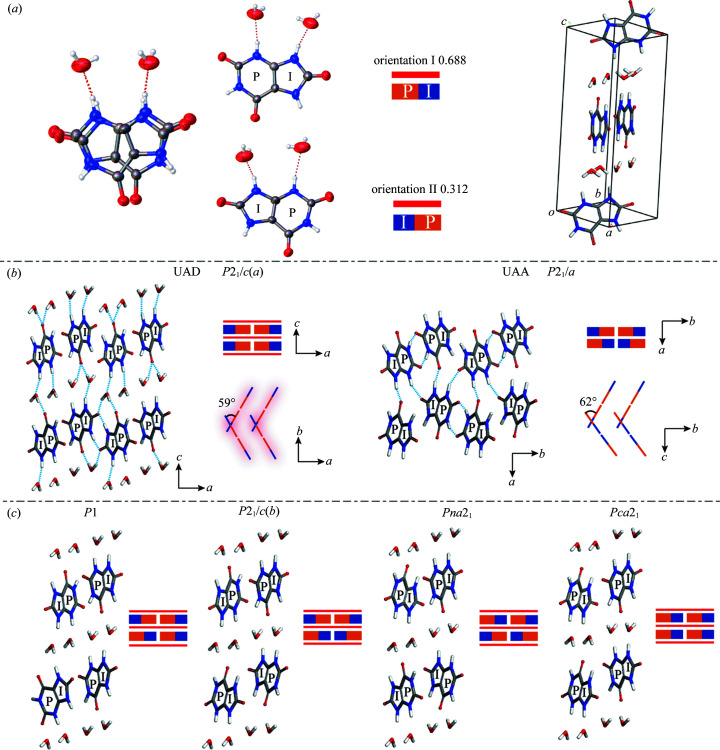
Orientational disorder and potential crystal-packing arrangements: (*a*) two distinct orientations (P-to-I *versus* I-to-P) of the UA molecule in the UAD unit cell with the chemical occupancy 0.688:0.312; (*b*) molecular packing of UAD (Parkin & Hope, 1998 ▸) and UAA (Ringertz, 1966 ▸); (*c*) molecular packing of potential disordered packing arrangements of UAD. Carbon, nitro­gen, oxygen and hydrogen atoms are marked in gray, blue, red and white, respectively.

Therefore, we examined and structurally resolved the MC by SCXRD. The crystallographic data and refinement details are presented in Table S3. It was found that MC crystallizes in the space group *P*2_1_/*c* including one UA and two water molecules, which is consistent with previous reports (Parkin & Hope, 1998 ▸; Artioli *et al.*, 1997 ▸; Thornton *et al.*, 2020 ▸; Izatulina *et al.*, 2019 ▸). But here we also found the presence of disordered UA molecules in the crystal structure with the chemical occupancy 0.688:0.312, as shown in Fig. 7 ▸(*a*), where one UA molecule (P-to-I orientation) is rotated ∼180° along the vertical direction to adopt another I-to-P orientation. When UA molecules are stacked with only P-to-I orientation to form ‘perfect’ UAD packing, the pyrimidine (marked as P) and imidazole (marked as I) in the adjacent purine rings are linked by the I–P⋯P–I⋯I–P⋯P–I stacking sequence to form 1D hydrogen-bonded ribbons. The neighboring purine layers stack in parallel and are rotated 59° to form 2D sheets in the *ac* plane, and the water layers are separated by the adjacent UA layers via hydrogen bonds with the UA molecules (Parkin & Hope, 1998 ▸) [Fig. 7 ▸(*b*), left]. This molecular arrangement is similar to the packing sequence of UAA [Fig. 7 ▸(*b*), right] (Ringertz, 1966 ▸). In the crystal structure of the UAA phase, the UA molecules are linked to their neighbors by hydrogen bonds to form 1D ribbons, and the adjacent ribbons stack in parallel but are rotated 62° to form 2D sheets in the *ab* plane. Yet the distance between the UA ribbons is smaller because no water layers are present. Also, the UA molecules in parallel ribbons (*ab* plane for UAD, *bc* plane for UAA) stack via π–π interactions (Figs. S12 and S13).

When UA molecules adopt two distinct orientations in the crystal structure, they can form five possible packings. We built a 3 × 3 × 1 supercell and the UA molecules stack in I-to-P and P-to-I orientations with the chemical occupancy 0.688:0.312 (Fig. S14), and four disordered arrangements are summarized in Fig. 7 ▸(*c*). The main difference among these disordered packing arrangements is the ring sequences of one 1D hydrogen-bonded ribbon and neighboring purine layers, which may be classified into four crystal systems, *P*1, *P*2_1_/*c*, *Pca*2_1_ and *Pna*2_1_, consistent with the classification by Demichelis (2020 ▸). Furthermore, we calculated the lattice energy to examine the stability of the five potential structures of UAD (Spackman, 2018 ▸; Maschio *et al.*, 2011 ▸; Dunitz & Gavezzotti, 2012 ▸; Abramov, 2017 ▸; van Eijck & Kroon, 1997 ▸; Tsuzuki *et al.*, 2010 ▸) (see supporting information for details), and the results are given in Table 2 ▸. As expected, in the molecular packing with no disordered UA molecules, *P*2_1_/*c*(*a*) presents the minimum lattice energy calculated, verifying the most stable crystal structure of UAD. Other packing structures have energies (absolute value) higher than *P*2_1_/*c*(*a*) calculated by DFT and CE-B3LYP, indicating they are metastable and easier to reconstruct. The electronic energy of molecule stacking (Demichelis, 2020 ▸) also demonstrates the metastable nature of these packing arrangements with energies higher than UAD *P*2_1_/*c*(*a*) by 1–6 kJ mol^−1^ (Table 2 ▸). Moreover, the interaction energy of UA with surrounding water molecules in four UAD structures was calculated using *CrystalExplorer17* (Table S6) (Maschio *et al.*, 2011 ▸; Spackman *et al.*, 2021 ▸). The average interaction energy of each water molecule in *P*2_1_/*c*(*a*), *P*2_1_/*c*(*b*), *Pca*2_1_ and *Pna*2_1_ packing are −50.99, −45.58, −44.97 and −45.1 kJ mol^−1^, respectively. The results reveal that water molecules in disordered packing structures (*e.g.* *P*2_1_/*c*(*b*), *Pca*2_1_ and *Pna*2_1_) are more prone to be removed from the network during dehydration. Overall, these results suggest that the presence of microscopic defects such as orientational disorder accelerates molecular reconstruction in the course of UAD dehydration and thus promotes solid-state phase transitions inside the crystal (which apparently presents as the core UAA phase).

**Figure 8:**
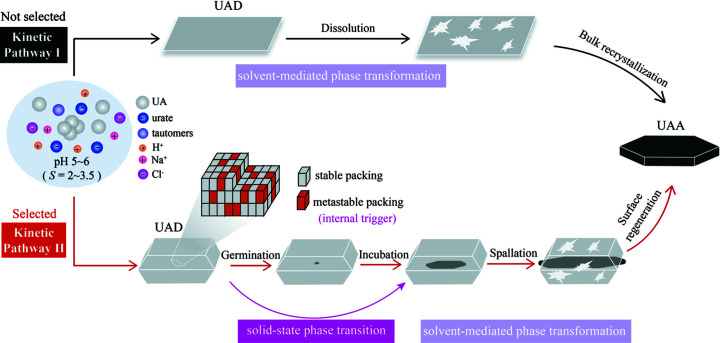
Proposed mechanism of the shape-preserved core-shell phase transition.

Collectively, we have demonstrated the unique phase-transition pathway from UAD to UAA, leading to the formation of an unconventional core-shell structure, as shown in Fig. 8 ▸. Unlike the conventional solvent-mediated phase transformation from a metastable to more stable phase (kinetic pathway I), this unusual ‘core-shell’ phase-transition behavior exhibits the onset dehydration transition of UAD occurring in the core of crystals, followed by local dissolution and recrystallization of the UAA phase on the surface, and thus adopts a combined solid-state and solvent-mediated phase transition (selected pathway II). One of the key features of the ‘core-shell’ phase transition is the intriguing formation of a new phase in the crystal interior via a solid-state dehydration phase transition. Within a certain crystallization regime, the thick-plate type-II crystal was formed in the presence of microscopic defects (*i.e.* orientational disorder) in the crystal lattice which functions as an ‘internal trigger’ for kinetic pathway selection. In an asymmetric unit, the UA molecule adopts two distinct orientations with the ratio 688:312 to form metastable disordered packing structures, lowering the energy barrier for dehydration and accelerating the solid-state dehydration phase transition. Upon nucleation of the new UAA phase in the crystal core or interior, the continuous spreading of the core phase accompanies the gradual depletion of mother shell crystals, leading to the formation of core-shell crystals (germination and incubation stages). As the core phase protrudes from the shell ends and is exposed in solution, the shell surface starts to dissolve locally with concomitant recrystallization of the core UAA phase via solvent-mediated phase transformation (spallation stage). Finally, the core UAA phase continuously grows and gradually develops the smooth facets (regeneration stage).

## 3. Conclusions

Herein, we discovered a unique phase-transition pathway which forms the core-shell structure with shape-preserved crystal evolution from the UAD to the UAA phase. The unusual pathway of phase transition was internally triggered through a solid-state dehydration phase transition occurring in the crystal core, followed by surface dissolution of the ‘mother’ shell UAD crystal and recrystallization of the ‘daughter’ core UAA phase. The crystal shape was largely maintained over the course of the phase transition. Furthermore, we found that UAD crystals had undergone the core-shell transition pathway have a strong association with crystallization supersaturations and the thickness of crystals.

The solid-state phase transition occurring prior to solvent-mediated phase transformation is unexpected in solution and related to orientational disorder and the high-energy orientation of UA molecules presumably due to crystallization interrupted by urate tautomerism. The disordered packing arises from the energy of the crystal lattice and lowers the energy barrier for the solid-state dehydration transition. To our knowledge, we have demonstrated, for the first time, an unusual core-shell phase-transition pathway in solution which combines a solid-state phase transition and a solvent-mediated phase transformation. Our findings also prove that a solid-state phase transition is not necessarily slower than a solvent-mediated phase transformation in solution. More broadly, our findings may provide a new approach that strategically imports internal defects to tailor the phase transition or crystallization pathways for various applications in crystal engineering.

## 4. Related literature

The following references are cited in the supporting information: Chih *et al.* (2016 ▸); Ding *et al.* (2017 ▸); Dolomanov *et al.* (2009 ▸); Mentasti *et al.* (1983 ▸); Sheldrick (2015 ▸); Thomas *et al.* (2018 ▸).

## Supplementary Material

Crystal structure: contains datablock(s) uricaciddihydrate. DOI: 10.1107/S2052252522011034/yc5039sup1.cif

Structure factors: contains datablock(s) uricaciddihydrate. DOI: 10.1107/S2052252522011034/yc5039sup2.hkl

Supporting information file. DOI: 10.1107/S2052252522011034/yc5039sup3.mp4

Supporting information file. DOI: 10.1107/S2052252522011034/yc5039sup4.mp4

Supporting figures and tables. DOI: 10.1107/S2052252522011034/yc5039sup5.pdf

CCDC reference: 2163198

## Figures and Tables

**Table 1:** Comparison of the time of onset phase transition for type-II and type-III crystals in air

Temperature (°C)	Relative humidity (%)	Type II		Type III	
		Surface	Core	Surface	Core†
22	12	59 h	>240 h	54 h	–
	44	92 h	>240 h	75 h	–
	75	>240 h	>240 h	>240 h	–
30	12	14 h	20 h	14 h	–
	44	45 h	24 h	23 h	–
	75	128 h	25 h	50 h	–
37	12	10 h	8 h	11 h	–
	44	36 h	12 h	24 h	–
	75	31 h	13 h	21 h	–

† No core phase detected.

**Table 2:** Lattice energies of five potential packing arrangements (*P*2_1_/*c*(*a*), *P*2_1_/*c*(*b*), *P*1, *Pca*2_1_ and *Pna*2_1_) of UAD and crystal packing of UAA

Crystal form	Packing†	Electronic energy† (kJ mol^−1^)	Lattice energy‡ (kJ mol^−1^)	Lattice energy§ (kJ mol^−1^)
UAD	*P*2_1_/*c*(*a*)	0.0	−268.46	−367.39
	*P*2_1_/*c*(*b*)	1.4	−246.14	−363.36
	*P*1	4.5	−234.33	–
	*Pca*2_1_	2.1	−72.20	−349.59
	*Pna*2_1_	6.1	−231.70	−359.75
UAA	*P*2_1_/*a*	–	−127.34	−210.24

† Referenced in the work by Demichelis (2020 ▸).

‡ Lattice energies calculated by DFT.

§ Lattice energies estimated using CE-B3LYP in *Crystalexplorer17*.
