# Supplementary material for: Unusual shape-preserved pathway of a core-shell phase transition triggered by orientational disorder
Source: IUCrJ. 2023 Jan 1;10(Pt 1):38–51. doi: 10.1107/S2052252522011034 (PMC9812221; doi:10.1107/S2052252522011034)
Supplement: Supplementary file 5 [file m-10-00038-sup5.pdf]

IUCrJ

Volume 10 (2023)

Supporting information for article:

Unusual shape-preserved pathway of a core-shell phase transition triggered by orientational disorder

Mengya Li, Weiwei Tang and Junbo Gong

S1. Experimental

S1.1. Solution and crystal preparation.

6.5 mM uric acid (UA, Sigma-Aldrich, 99 %) solution was prepared by dissolving 109.27 mg uric acid powders in 100 ml deionized water containing 1.25 mL 1 M NaOH (aq). The solution was then heated to 37 °C, and 818.6 mg NaCl powder (Aladdin, 99.5 %) was added into solution to obtain 0.14 M Na^{+}. The pH of solution was adjusted to pH=5.50 (s.d.: ±0.01) by addition of appropriate amounts of 10 % mass fraction HCl (aq). Certain amounts of solution were taken out by a 10 mL syringe, filtered out using pre-heated filter membrane (Φ 22 μm), and transferred into a glass bottle. The glass bottle was placed in a 37 °C oven to cultivate uric acid dihydrate (UAD) single crystals.

S1.2. *In situ* characterization of crystallization and crystal morphology evolution.

The crystallization of UAD crystals was monitored under a custom-built, inverted optical microscope. A supersaturated solution of 6.5 mM UA, prepared above, was injected to a quartz colorimeter (length 50 mm, width 10 mm and depth 2 mm) and sealed by the cap and parafilm. The quartz colorimeter was then quickly transferred into a glass crystallizer, which was kept at 37 °C by the water circulating bath system. The inverted microscope (Olympus) was employed to observe time-elapsed morphology evolution of crystals.

S1.3. Analytical techniques.

Scanning electron microscope (SEM, TM3000) was conducted under vacuum with an accelerating voltage of 15.00 kV. SEM was employed to characterize the morphology and microstructure of sampled crystals at different crystallization stages. Field emission transmission electron microscope (TEM, Tecnai G2 F20) with selected area electron diffraction (SAED) was performed to identify structure and crystallinity of crystals harvested at different stages. The measurement was carried out on an accelerating voltage of 200 kV. Powder X-ray diffraction (PXRD) patterns of crystal samples at different crystallization stages were measured by Rigaku X-Ray Diffractometer with Cu Kα radiation (1.5405 Å) and worked at 100 mA current and 40 kV voltage. All samples were scanned from 2° to 40° with

a scanning rate of 8°/min. Furthermore, fourier transform infrared spectra (FTIR, Bruker Alpha FTIR-ATR, German) were collected in the spectral range of 4000-400 cm^{-1} with 4 cm^{-1} resolution and with an average of 32 scans per spectrum. Single crystal X-ray diffraction (SCXRD) measurements were performed on a Rigaku XtaLAB Synergy HyPix diffractometer using Mo-Kα radiation (λ = 0.71073 Å). Data collection was carried out at 298.43 K, and Olex2 software and SHELXT were used for structure solution and refinement (Dolomanov *et al.*, 2009; Sheldrick, 2015).

S1.4. Spatial-resolved 2D Raman spectroscopy.

Micro-Raman spectrometer with a 632 nm laser (RENISHAW invia reflex) was utilized to characterize local structure of single crystals sampled at different crystallization stages. Each spectrum was collected in the spectral range of 100-2000 cm^{-1} at a resolution of 1 cm^{-1} with the number of scans at least 30 times.

S1.5. Urate concentration and pH measurements.

The microplate reader (Spectra Max M_2^e) was used to measure the time-elapsed change of urate concentration in solution during crystallization. Crystallization was performed from a supersaturated solution of 6.5 mM UA at pH 5.50 and 37 °C. Upon nucleation, about 0.5 mL supernatant was taken out at a certain time period and filtered by 1mL syringe with filter membrane and then transferred into centrifuge tube. The clear solution was further diluted to measure the intensity of absorption peak at 290 nm by microplate reader. The standard curve for concentration measurement was constructed in advance. Concentration measurement at each time point was performed in triplicate. Solution pH was measured by pH meter (FiveEasy Plus, Mettler-Toledo, Switzerland) over the course of crystallization. The solubilities of UAA and UAD phases were measured using a dynamic method reported in literature (Chih *et al.*, 2016; Mentasti *et al.*, 1983; Ding *et al.*, 2017) as a function of pH.

S1.6. Thermal analyses.

Thermogravimetric analyzer (TGA/DSC, Mettler-Toledo, Switzerland) was used to characterize thermodynamic property of crystals obtained in different crystallization stages,

and crystal powders of UAD and uric acid anhydrate (UAA) were prepared followed by a reported method in literature (Zellelow *et al.*, 2010; Zellelow *et al.*, 2010). The measurement was carried out under the protection of nitrogen flow at the temperature ranging from 30 °C to 450 °C with a heating rate of 5 °C/min. The amount of sample used was about 5-10 mg. The dehydration process of crystals were investigated by differential scanning calorimetry (DSC, Mettler-Toledo, Switzerland) at heating rates of 2 K/min, 5 K/min, 10 K/min, 15 K/min, and 20 K/min. The amount of sample used was about 5-10mg.

S1.7. Phase transition in air.

Different types of crystals were prepared and dispersed on glass slides. Nine specimen bottles were divided into three groups to hold saturated NaCl (relative humidity 75 %), K_2CO_3 (relative humidity 44 %) and LiCl (relative humidity 12 %) solution. The glass slide was mounted on the holder in the specimen bottles, and the specimen bottle with glass side sample was placed at room temperature (22 °C) or thermotank (30 °C, 37 °C). The morphology of crystals placed in air at different temperatures and relative humidity was monitored by optical inverted-microscope over time. We observed the appearance of the core phase in mother crystal and quantified the time of onset phase transition of the core phase of mother crystal. The dimension of core phase of core-shell crystal was measured over time, and the turning point of the dimension-time curve was defined as the time of onset phase transition of the core phase of core-shell crystal. In addition, the appearance time of spots and cracks on the surface of crystals was defined as the transition time of crystal surface. More than ten individual crystals were statistically counted under each experimental condition.

S1.8. Lattice energy calculation.

Lattice energy based on DFT calculations were carried out in Material Studio using $DMol^3$ module. In the $DMol^3$ method (Tsuzuki *et al.*, 2010), the physical wave functions are expanded in terms of accurate numerical basis sets. We used the double numerical-quality basis set with polarization functions (DNP) for geometry optimizations. The triple-numerical quality basis set with polarization functions (TNP) was used for energy calculations, unless noted otherwise. The PW91 functional

was employed, and the positions of atoms in the crystal were optimized by periodic DFT calculations. The experimental lattice parameters were fixed during the optimization. The lattice energy was then calculated using eq 1:

$$E_{lattice} = \frac{1}{Z} E_{crystal\ cell} - E_{mono} \qquad (1)$$

where Z is the number of molecules in on unit cell, $E_{crystal\ cell}$ is the energy of a crystal per unit cell calculated by the periodic DFT method from the optimized crystal structure. E_{mono} is the energy for an isolated molecule. Here, the stoichiometry of uric acid and water in UAD is 1:2, we set one uric acid and two water as one "monomer", and set Z as 4. The calculated lattice energies of different packing arrangements of UAD and UAA are shown in Table S4.

CE-B3LYP lattice energies are computed by direct summation of interaction energies in CrystalExplorer17 over molecules B interacting with a central molecule A until lattice energy $E_{lattice}$ is converged to better than 1 kJ/mol, using a cutoff based on the separation of molecular centroids, R_{AB} (Spackman *et al.*, 2021; Thomas *et al.*, 2018; Spackman, 2018). As described in detail elsewhere, for unit cells with nonzero dipole moments (i.e., polar space groups such as $P2_1$, $Pna2_1$, and $Fdd2$) the lattice summation above is not representative of the entire crystal, and an additional consideration, cell dipole energy, is required (Maschio *et al.*, 2011; Dunitz & Gavezzotti, 2012; Abramov, 2017; van Eijck & Kroon, 1997). The lattice energy of UAD and UAA was calculated following eq 2:

$$E_{lattice} = \frac{1}{2} \sum_{R_{AB}<R} E_{tot}^{AB} + E_{cell\ dipole} = \frac{1}{2} \sum_{R_{AB}<R} E_{tot}^{AB} - \frac{2\pi p_{cell}^2}{3ZV_{cell}} \qquad (2)$$

where the second term is the cell dipole energy correction. p_{cell} is the magnitude of the dipole moment of the unit cell (obtained here as the vector sum of molecular dipole moments), V_{cell} its volume, and Z is the number of molecules in on unit cell. The cell dipole energies for polar space groups and lattice energies of different packing arrangements of UAD and UAA are shown in Tables S5 and S6, respectively.

S1.9. Atomic force microscope (AFM) measurement.

A dimension icon atomic force microscope (Bruker, Germany) was employed to examine the surface topographical features of different types of crystals. AFM mages were collected in ScanAsyst mode using ScanAsyst-Air probes (silicon nitride). We painted the heated mixture of the epoxy resin and curing agent on the glass slide and placed it under room temperature about ten minutes to form a smooth substrate. The crystals were adhered on the smooth substrate, and then were scanned at a rate of 1 Hz with 256 lines.

S2. Activation energy calculation

The influence of various heating rates under non-isothermal crystallization process on the activation energy can be described using the Kissinger-Akahira-Sunrose (KAS) method and the Flynn-Wall-Ozawa (FWO) method (Starink, 2003; Ozawa, 1992). The KAS method is based on the well-known Kissinger equation and has the form shown in eq 3:

$$\ln(\frac{\beta}{T_p^2}) = \ln(\frac{AR}{E_a g(\alpha)}) - (\frac{E_a}{RT_p}) \qquad (3)$$

where β represents the heating rate, T_p is the onset temperature of the transition in K, E_a is the activation energy of phase transition in J/mol, A is the pre-exponential factor in s^{-1}, R is the general gas constant (8.314 J/mol/K), g(α) is a mechanism-related function. The FWO method is in fact a "model free" method which involves measurement of the temperatures corresponding to fixed values of α from experiments at different heating rates β, and as shown in eq 4:

$$\log\beta = \log(\frac{AE_a}{Rg(\alpha)}) - 2.315 - 0.4567(\frac{E_a}{RT_p}) \qquad (4)$$

DSC curves of the dehydration of different types of crystals at different heating rates are shown in Figures S10 and S11, respectively. The measurement was carried out under the protection of nitrogen flow at the temperature ranging from 30 ℃ to 450 ℃ with a heating rate of 5 ℃/min. The amount of sample used was about 5-10 mg. The dehydration process of crystals were investigated by differential scanning calorimetry

(DSC, Mettler-Toledo, Switzerland) at heating rates of 2 K/min, 5 K/min, 10 K/min, 15 K/min, and 20 K/min. The amount of sample used was about 5-10mg.

S3. Supporting Figures

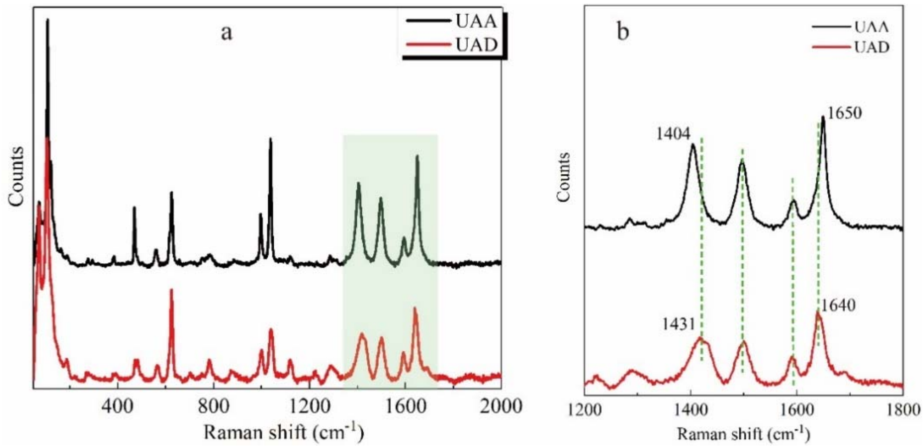

Figure S1 Comparisons of Raman spectra of (a) UAD and UAA, and (b) the enlarged 1200-1800cm^{-1} range of Raman spectra showing distinct differences between UAD and UAA phases.

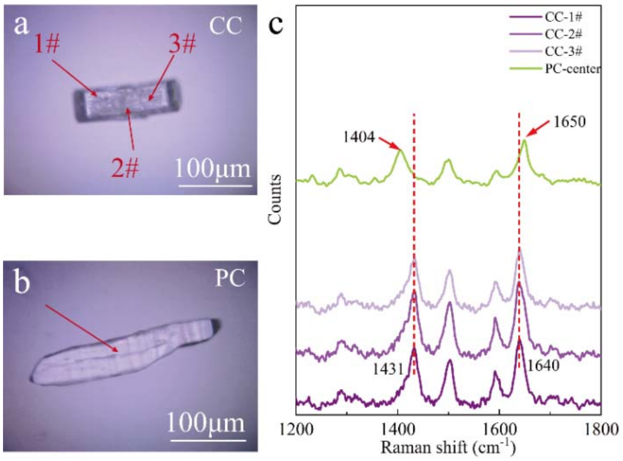

Figure S2 Optical micrographs of (a) CC and (b) PC, as well as (c) their Raman spectra.

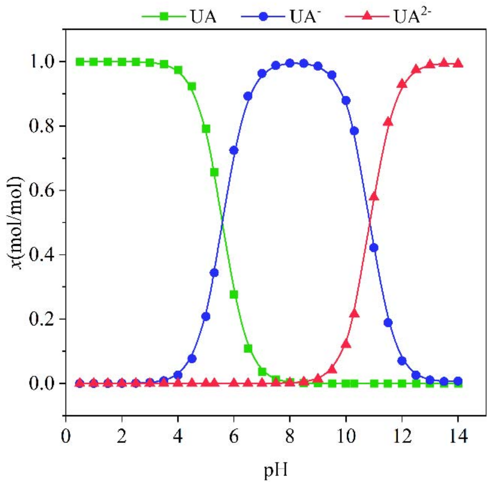

Figure S3 UA speciation as a function of pH in the presence of 0.14 M Na^+ in solution.

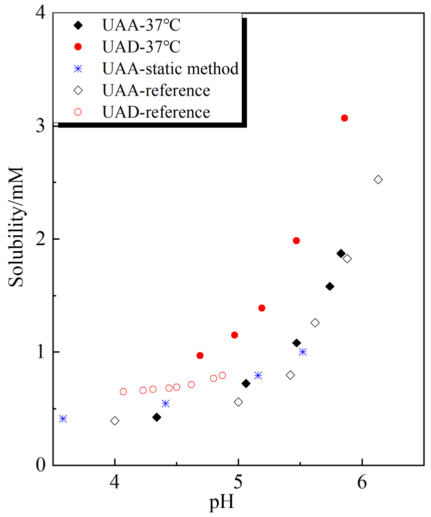

Figure S4 Solubility of UAA and UAD determined in this work and comparisons with reported values in literature (Chih *et al*., 2016; Mentasti *et al*., 1983; Ding *et al*., 2017).

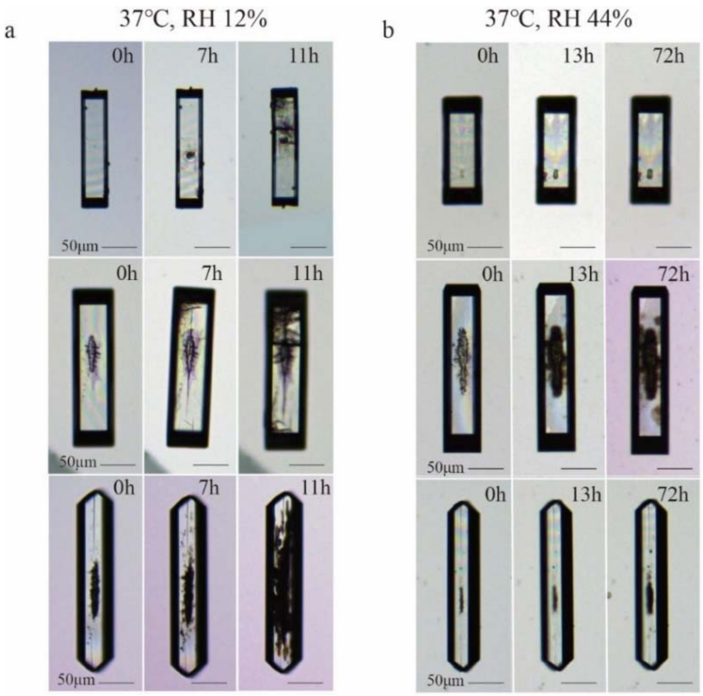

Figure S5 Morphology evolution of MC and CS placed in air under 37°C with RH (a) 12% and (b) 44%.

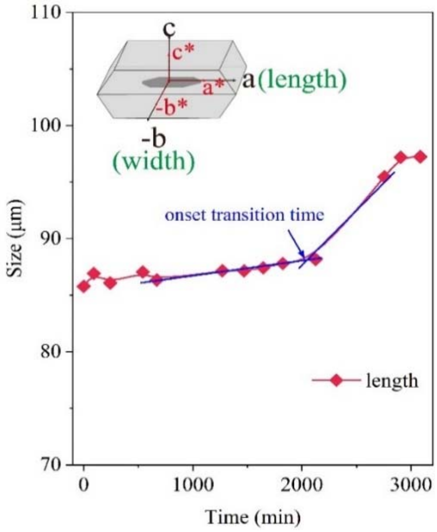

Figure S6 Time-elapsed size evolution of the core phase along a^*-axis in CS crystal. Blue arrow defines the onset time of phase transition.

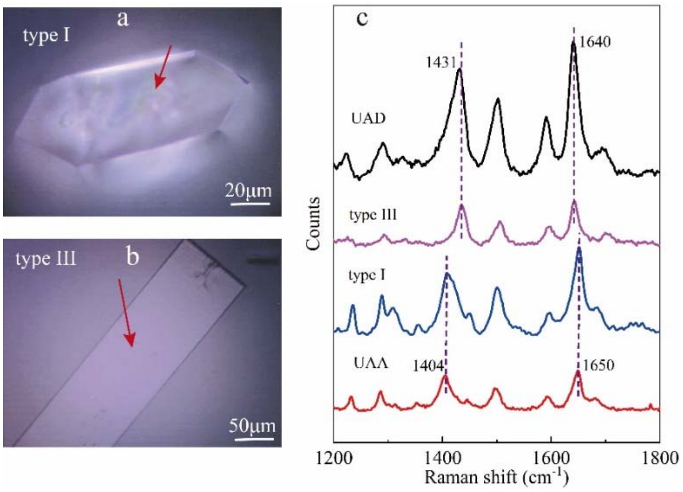

Figure S7 Optical micrographs (a, b) and Raman spectra (c) of type I and III crystals obtained from different supersaturated solutions.

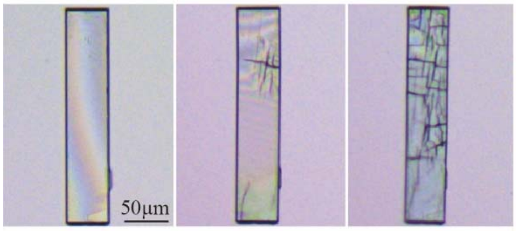

Figure S8 Morphology evolution of type III placed in air under 37°C with RH 12%.

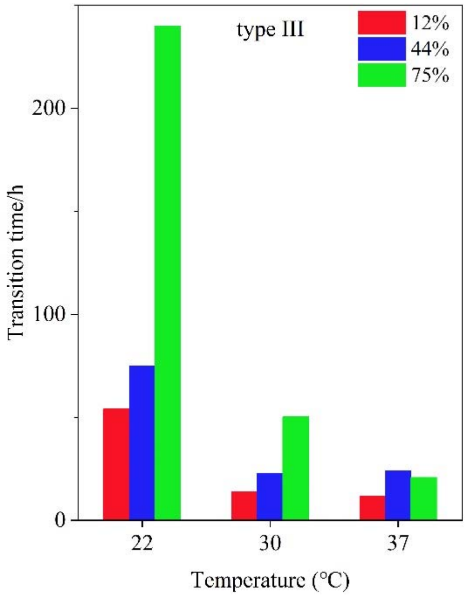

Figure S9 The initial transition time of type III crystal placed in air at different temperatures and relative humidity.

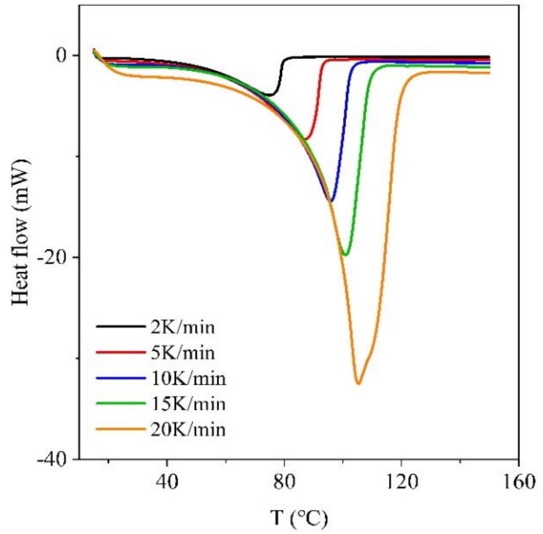

Figure S10 DSC curves collected simultaneously in the dehydration of type II crystals at different heating rates.

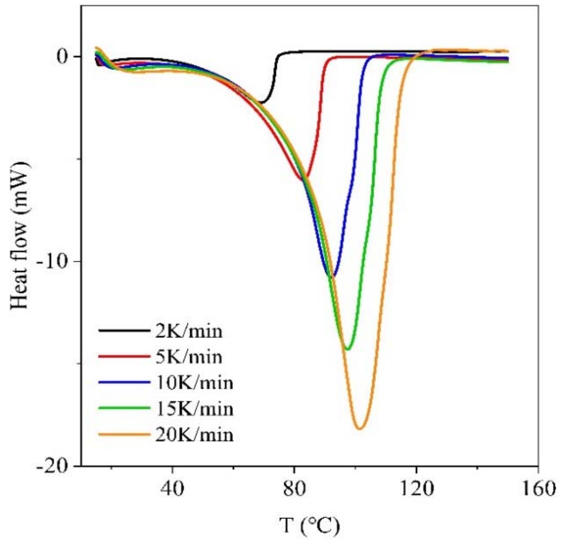

Figure S11 DSC curves collected simultaneously in the dehydration of type III crystals at different heating rates.

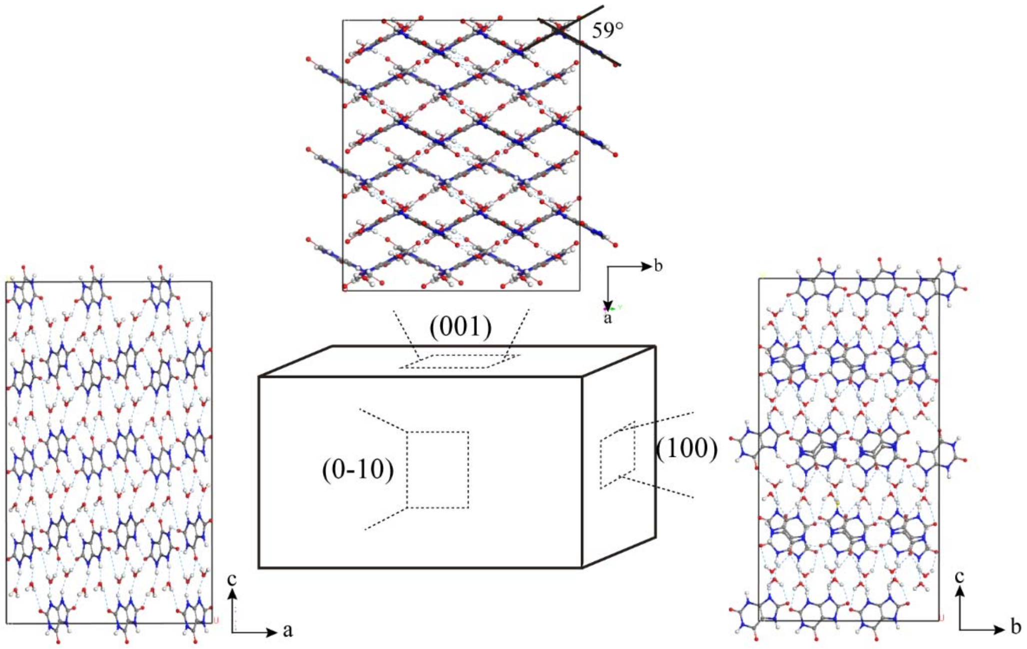

Figure S12 Molecular arrangements and crystal packing of UAD viewed down (001), (100), and (0-10) planes.

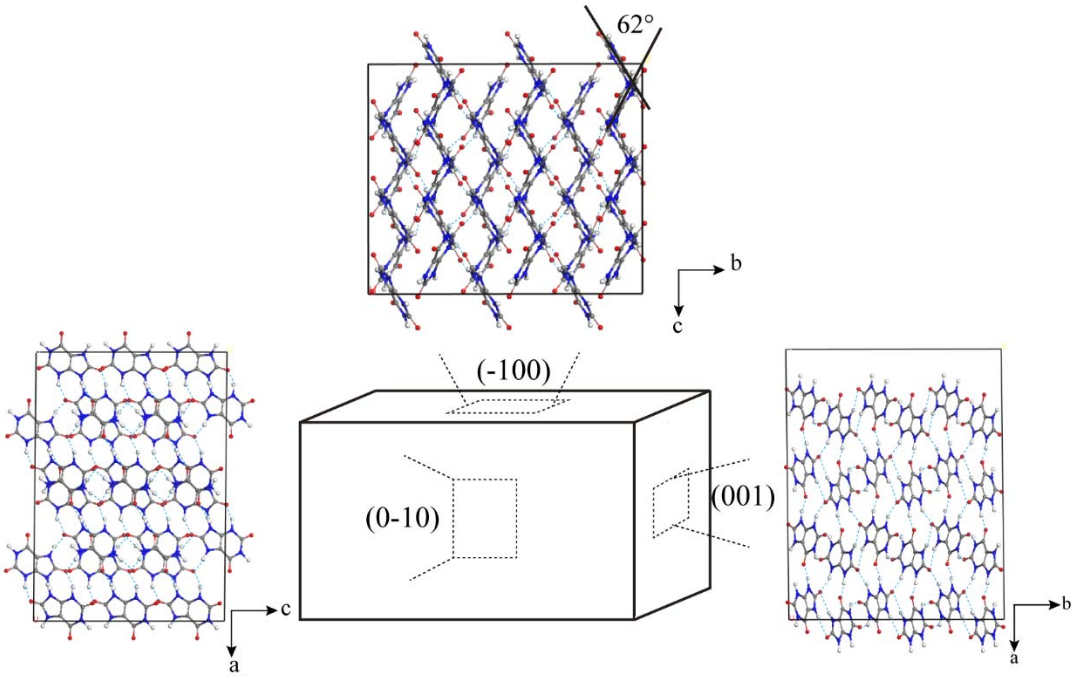

Figure S13 Molecular arrangements and crystal packing of UAA viewed down (001), (-100), and (0-10) planes.

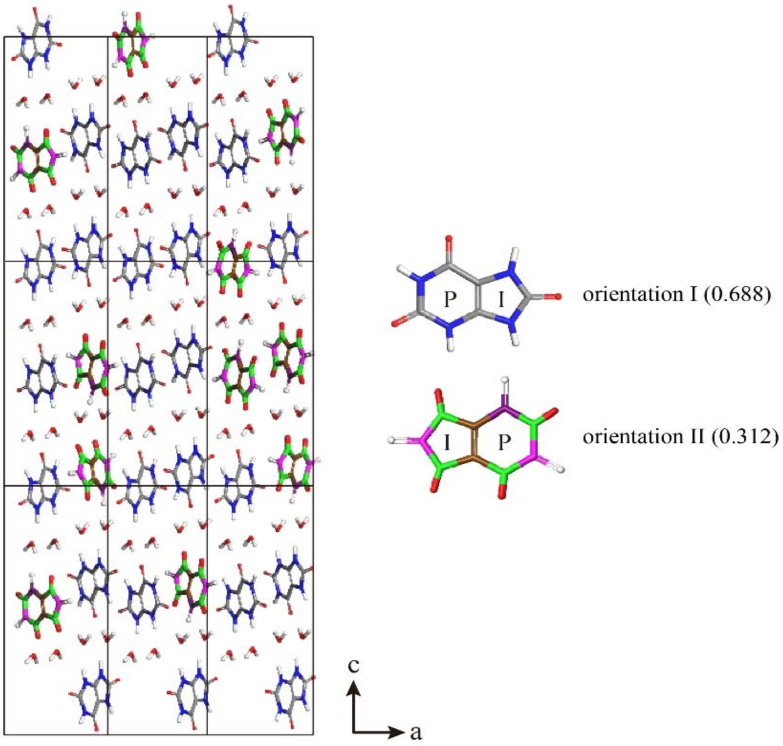

Figure S14 The supercell (3×3×1) of UAD crystal structure with disordered stacking. The pyrimidine ring is marked as P and imidazole ring is marked as I in the adjacent purine rings.

S4. Supporting Tables

Table S1 Crystals obtained from different initial pH and UA concentrations.

pH \ UA	3.5mM	4.5mM	5.5mM	6.5mM	7.5mM	8.5mM	9.5mM
**5.10**	II	II	II+III[a]	III	III	III	III
**5.30**	II	II	II	II+III[a]	II+III[b]	III	III
**5.50**	I	II	II	II	II+III	II+III[b]	II+III[b]
**5.70**	I	I	II	II	II	II	II
**5.90**	-[c]	IV	IV	IV	IV	IV	IV

[a] represents type II crystals predominant; [b] represents type III crystals predominant; [c] represents no crystal is obtained within a week.

Table S2 Corresponding initial supersaturations of UA at different pHs and concentrations.

pH \ UA	3.5mM	4.5mM	5.5mM	6.5mM	7.5mM	8.5mM	9.5mM
**5.10**	2.70	3.47	4.24	5.01	5.78	6.55	7.32
**5.30**	2.23	2.87	3.51	4.15	4.78	5.42	6.06
**5.50**	3.02[a]	2.31	2.82	3.34	3.85	4.36	4.88
**5.70**	2.28[a]	2.93[a]	2.21	2.62	3.02	3.42	3.82
**5.90**	-[b]	-[c]	-[c]	-[c]	-[c]	-[c]	-[c]

[a] represents the initial supersaturation relative to UAA phase; [b] represents no crystal obtained within a week; [c] represents the formation of MSUM crystals.

Table S3 Crystallographic data and refinement details of type II crystal (UAD) solved in this study.

Formula	$C_5H_8N_4O_5$
Formula weight	204.15
Temperature/K	298.43(10)
Crystal system	monoclinic
Space group	$P2_1/c$
a/Å	7.4040(4)
b/Å	6.3496(3)
c/Å	17.5648(10)
α/°	90
β/°	89.977(5)
γ/°	90
Volume/$Å^3$	825.76(8)
Z	4
$\rho_{calc}g/cm^3$	1.642
μ/mm^{-1}	0.147
F(000)	424.0
Crystal size/mm^3	0.15× 0.1 × 0.08
Radiation	Mo Kα (λ = 0.71073)
2Θ range for data collection/°	4.638 to 61.692
Index ranges	$-10 \leq h \leq 10$, $-7 \leq k \leq 8$, $-20 \leq l \leq 23$
Reflections collected	11931
Independent reflections	2197 [R_{int} = 0.0416, R_{sigma} = 0.0376]
Data/restraints/parameters	2197/224/231
Goodness-of-fit on F^2	1.071
Final R indexes [I>=2σ (I)]	R_1 = 0.0533, wR_2 = 0.1325
Final R indexes [all data]	R_1 = 0.0858, wR_2 = 0.1481
Deposition Number in CCDC	2163198

Table S4 Calculated lattice energies of different possible packing arrangements of UAD and UAA.

Sample	Space group	E_{cell} Ha	$E_{\text{uric acid}}$ Ha	E_{water} Ha	$E_{lattice}$ kJ/mol
UAD	$P2_1c(a)$	-15.905	-3.114	-0.38	-268.46
	$P2_1c(b)$	-15.871	-3.114	-0.38	-246.14
	$P1^c$	-15.917	-3.130	-0.38	-234.33
	$Pca2_1$	-15.610	-3.115	-0.38	-72.20
	$Pna2_1$	-15.909	-3.129	-0.38	-231.70
UAA	$P2_1a$	-12.650	-3.114	-	-127.34

Table S5 Cell dipole energies for polar space groups[a]. Cell dipole moments are obtained from the vector sum of B3LYP/6-31G(d,p) molecular dipoles.

Sample	Space group	Z	V_{cell}/Å^3	P_{cell}/D	$E_{cell\ dipole}$/kJ/mol
UAD	$P2_1c(a)$	4	825.76	5.188	-1.03
	$P2_1c(b)$	4	801.569	3.434	-0.46
	$Pca2_1$	4	803.718	2.346	-0.22
	$Pna2_1$	4	776.344	8.773	-3.13
UAA	$P2_1a$	4	602.94	3.886	-0.79

[a] Cell dipole energy is given by the expression $E_{cell\ dipole} = \frac{-2\pi p_{cell}^2}{3ZV_{cell}} = -126.13\frac{(p_{cell}/D)^2}{Z(V_{cell}/\text{Å}^3)}kJ/mol$

Table S6 Lattice energies of different possible packing arrangements of UAD and UAA calculated by CrystalExplorer17.

Sample	Space group	$E_{uric\ acid}$ kJ/mol	E_{water} kJ/mol	$E_{cell\ dipole}$ kJ/mol	$E_{lattice}$ kJ/mol
UAD	$P2_1c(a)$	-264.38	-50.99	-1.03	-367.39
	$P2_1c(b)$	-271.74	-45.58	-0.46	-363.36
	$Pca2_1$	-259.43	-44.97	-0.22	-349.59
	$Pna2_1$	-266.42	-45.1	-3.13	-359.75
UAA	$P2_1a$	-209.45	-	-0.79	-210.24

S5. Supporting Movies

Movie S3 The crystal morphology evolution of type II crystal in supersaturated solution (6.5 mM UA, pH 5.5, 37°C) under inverted-microscope. The movie is composed of 62 optical micrographs at an interval of 2 hrs for a total imaging time of 124 hours.

Movie S4 The crystal morphology evolution of type III crystal in supersaturated solution (6.5 mM UA, pH 5.1, 37°C) under inverted-microscope. The movie is composed of 32 optical micrographs at an interval of 2 hrs for a total imaging time of 64 hours.

References

Abramov, Y. A. (2017). *Cryst. Growth Des.* **17**, 2873-2880.

Chih, M. H., Lee, H. L. & Lee, T. (2016). *CrystEngComm* **18**, 290-297.

Ding, Z., Zhang, H., Han, D., Zhu, P., Yang, P., Jin, S., Li, M. & Gong, J. (2017). *J. Chem. Eng. Data* **62**, 3929-3937.

Dolomanov, O.V., Bourhis, L.J., Gildea, R. J., Howard, J.A.K. & Puschmann, H. (2009). *J. Appl. Cryst*. **42**, 339-341.

Dunitz, J. D. & Gavezzotti, A. (2012). *J. Phys. Chem. B*. **116**, 6740-6750.

Maschio, L., Civalleri, B., Ugliengo, P. & Gavezzotti, A. (2011). *J. Phys. Chem. A*. **115**, 11179-11186.

Mentasti, E., Rinaudo, C. & Boistelle, R. (1983). *J. Chem. Eng. Data* **28**, 247-251.

Ozawa, T. (1992). *Thermochimica Acta*. **203**, 159-165.

Sheldrick, G.M. (2015). *Acta Cryst*. **A71**, 3-8.

Spackman, M. A. (2018). *CrystEngComm* **20**, 5340-5347.

Spackman, P. R., Turner, M. J., McKinnon, J. J., Wolff, S. K., Grimwood, D. J., Jayatilaka, D. & Spackman, M. A. (2021). *J. Appl. Cryst.* **54**, 1006-1011.

Starink, M. J. (2003). *Thermochimica Acta.* **404**, 163-176.

Thomas, S. P., Spackman, P. R., Jayatilaka, D. & Spackman, M. A. (2018). *J. Chem. Theory Comput.* **14**, 1614-1623.

Tsuzuki, S., Orita, H., Honda, K. & Mikami, M. (2010). *J. Phys. Chem. B.* **114**, 6799-6805.

van Eijck, B. P. & Kroon, J. (1997). *J. Phys. Chem. B.* **101**, 1096-1100.

Zellelow, A. Z., Abiye, M., Fink, D. A., Ford, C. E., Kim, K.-H., Sours, R. E., Yannette, C. M. & Swift, J. A. (2010). *Cryst. Growth. Des.* **10**, 3340-3347.

Zellelow, A. Z., Cox, K. A., Fink, D. A., Ford, C. E., Kim, K.-H., Sours, R. E. & Swift, J. A. (2010). *Crys. Growth. Des.* **10**, 3348-3354.
